# Increased Thoracic Fluid as the Most Distinctive Cardiovascular Hemodynamic Alteration in Men with Prolactinoma

**DOI:** 10.3390/nu14245369

**Published:** 2022-12-17

**Authors:** Agnieszka Jurek, Paweł Krzesiński, Grzegorz Gielerak, Przemysław Witek, Grzegorz Zieliński, Anna Kazimierczak, Robert Wierzbowski, Małgorzata Banak, Beata Uziębło-Życzkowska

**Affiliations:** 1 Department of Cardiology and Internal Diseases, Military Institute of Medicine, 04-141 Warsaw, Poland; 2 Department of Internal Medicine, Endocrinology and Diabetes, Medical University of Warsaw, 03-242 Warsaw, Poland; 3 Department of Neurosurgery, Military Institute of Medicine, 04-141 Warsaw, Poland

**Keywords:** prolactin, prolactinoma, impedance cardiography, cardiovascular complications, hemodynamic dysfunction, arterial hypertension

## Abstract

Hyperprolactinemia in males with prolactin-secreting adenomas, or prolactinomas, may be associated with endothelial dysfunction and co-existing cardiovascular risk factors. As a noninvasive technique of assessing cardiac function, impedance cardiography (ICG) may be useful in the early detection of hemodynamic dysfunction. The aim of the present study was to analyze and compare the hemodynamic profiles of patients with prolactinoma versus controls. A total of 20 men with prolactinoma (PR group) (mean age 43 years) and 20 men from the control group (CG) were evaluated in this prospective, observational comparative clinical study. The study subjects were propensity score-matched in terms of clinical characteristics—age, mean blood pressure [MBP], arterial hypertension [AH] rates, and body mass index [BMI]. ICG assessments of hemodynamic profiles were conducted with the use of a *Niccomo*™ device and included stroke volume index (SI), cardiac index (CI), systemic vascular resistance index (SVRI), velocity index (VI), acceleration index (ACI), Heather index (HI), and thoracic fluid content (TFC). AH was well-controlled in both study groups (116/76 mmHg PR vs. 119/76 mmHg CG). In comparison with CG patients, ICG revealed PR group patients to have higher rates of high thoracic fluid content (TFC) (>35 1/kOhm; *p* = 0.035) and lower SI values (<35 mL/m^2^, *p* = 0.072). There was a convergent tendency towards lower values of other cardiac function parameters (SI, CI, VI, ACI, and HI). Prolactinoma-associated endocrine abnormalities are related to hemodynamic profile alterations, including higher rates of increased TFC and the risk of worsened cardiac function.

## 1. Introduction

Prolactin (PRL)-secreting tumors, or prolactinomas, are the most common pituitary adenomas. Excessive PRL secretion leads to hypogonadism in both sexes, with the type and severity of clinical symptoms depending on the sex, PRL levels, disease duration, tumor size, and the extent of hyperprolactinemia [1,2,3,4,5,6]. The data on the incidence of cardiovascular disease in patients with prolactinoma are scarce, but they do indicate an increased cardiovascular risk and mortality risk in this population, mainly due to concomitant metabolic disorders, hypercoagulability, and the early development of atherosclerosis [5,6,7,8,9,10,11,12,13,14,15]. The complex pathomechanisms that may influence the development of cardiovascular complications in patients with PR include vascular endothelial dysfunction with changes in peripheral vascular resistance, the co-occurrence of metabolic disorders, insulin resistance syndrome, and lipid metabolism disorders [9,10,11,12,13]. Secondary endothelial dysfunction and consequent impaired vasoconstriction play an important role in the development of cardiovascular disorders in patients with PR. In clinical studies, elevated PRL levels have been shown to be associated with a hypercoagulable state and an increase in pro-inflammatory biomarkers, including interleukin-6 [12,13]. Metabolic abnormalities and a hypercoagulable state are predictors of the development of preclinical atherosclerosis in this group of patients [12,13,14,15]. The hemodynamic profile of prolactinoma patients requires further study to identify those variables that significantly increase cardiovascular risk.

Mortality among adult males with prolactinoma was shown to be several times higher than in the general population and this was mainly due to cardiovascular complication [5,7,8]. The most common cardiovascular abnormalities observed in patients with prolactinoma include arterial hypertension (AH), left ventricular (LV) dysfunction, and endothelial dysfunction [9,10,11,16,17,18,19]. The AH in patents with prolactinoma is of secondary nature and may be associated with multiple metabolic disorders [8,9,19]. Control of AH in this group of patients can be difficult and requires personalized hypotensive therapy. Prolactinoma management seems to require combination treatment based on the assessment of cardiovascular risk factors as well as serum PRL levels [2,12]. Conventional methods of assessing hemodynamic function, such as systolic and diastolic blood pressure (BP) measurements with automatic arm sphygmomanometers, 24-h BP monitoring, and the assessment of AH-associated complications with standard echocardiography may have some limitations due to the incomplete depiction of pathophysiological changes. New non-invasive diagnostic methods are therefore being sought to allow early detection of abnormalities in patients with PR and increase the chance of targeted, optimal therapy resulting in reduced cardiovascular risk. Endocrine abnormalities in PR have a significant impact on cardiovascular function, leading to altered hemodynamic profiles in patients and increased cardiovascular risk.

Impedance cardiography (ICG), which is a modern, noninvasive, and well-established method for assessing hemodynamic function, may be useful in detecting early subclinical hemodynamic dysfunction in patients with prolactinoma [20,21,22,23,24]. The use of ICG in prolactinoma diagnostics and treatment offers a personalized assessment of each patient’s hemodynamic profile and a more complete insight into the prolactinoma’s pathophysiology. The aim of this paper was to analyze the hemodynamic profiles of prolactinoma patients and controls in order to identify prolactinoma-associated unfavorable hemodynamic findings.

## 2. Materials and Methods

### 2.1. Study Population

This prospective, observational study was a comparative analysis of 20 men with prolactinoma (PR group) with no clinically significant comorbidities and 20 men from the control group (CG), propensity score-matched in terms of clinical characteristics—age, mean blood pressure [MBP], arterial hypertension [AH] rates and body mass index [BMI].

The Ethical Committee of the Military Medical Institute-National Research Institute in Warsaw approved the study (No 76/WIM/2016) according to the principles of the Helsinki Declaration and the principles of Good Clinical Practice (GCP). Study subjects signed their written informed consent.

The PR group consisted of adult males diagnosed with a PRL-secreting pituitary adenoma based on the standard hormone and imaging criteria, i.e., the combination of clinical manifestations of hyperprolactinemia, increased serum PRL levels, and radiologic evidence of a pituitary tumor [1,25]. Functional hyperprolactinemia and treatment with drugs affecting the dopaminergic system were excluded in each case.

All PR group patients underwent comprehensive hormone level evaluation, including adrenocorticotropic hormone (ACTH), thyroid-stimulating hormone (TSH), luteinizing hormone (LH), and follicle-stimulating hormone (FSH) levels, and an assessment for co-existent carbohydrate disorders (impaired fasting glycaemia [IFG], impaired glucose tolerance [IGT], and type 2 diabetes mellitus [T2DM]). Since none of the patients in the PR group were taking any drugs that affect the function of the hypothalamus-pituitary-adrenal axis, their medical treatment had no effect on their hemodynamic function assessment results.

The 20 CG subjects were selected from 155 participants of the FINE-PATH study (Clinical Trials.gov. Identifier NCT01996085). This group comprised 120 individuals with AH treated for at least 12 months and 35 healthy individuals with no cardiovascular conditions or any other clinically significant internal medicine morbidities.

The exclusion criteria for the PR group and the control group were: coronary heart disease; heart failure (HF) with reduced and mid-range ejection fraction (EF), i.e., EF of <50%); history of pulmonary embolism, chronic obstructive pulmonary disease (COPD); history of stroke or transient ischemic attack; respiratory failure (arterial partial pressure of oxygen of <60 mmHg and/or increased partial pressure of carbon dioxide > 45 mmHg); condition after head injury; lack of informed consent; pregnancy; and any other patient conditions that would make it impossible to follow the study protocol.

### 2.2. Clinical Examination

All study subjects underwent a clinical examination for cardiovascular risk factors. This included a thorough history for any cardiovascular symptoms, comorbidities, smoking, carbohydrate metabolism disorders, lifestyle, and family history of cardiovascular disease. Measurements included anthropometric parameters (body weight, height, BMI), heart rate (HR), and office systolic and diastolic BP (SBP and DBP) values. Office BP measurements (OBPM) were performed according to the European Society of Cardiology (ESC) guidelines using an automatic sphygmomanometer (Omron M4 Plus, Kyoto, Japan) [26].

### 2.3. Impedance Cardiography

The ICG-measured hemodynamic parameters were assessed in patients with prolactinoma and in controls in a quiet setting, a sitting position, and in the presence of a trained nurse after a rest of at least 5-min in the morning. The ICG recordings obtained during 10-min examinations conducted under resting conditions in a horizontal position with a *Niccomo*™ device (Medis, Ilmenau, Germany) were analyzed in detail (*Niccomo Software*) in terms of the hemodynamic parameters, including SBP; DBP; HR; cardiac output (CO [mL*min^−1^]; stroke volume (SV [mL]); systemic vascular resistance (SVR [dyn*s*cm^−5^]); cardiac index (CI [mL*m^−2^*min^−1^]); stroke volume index (SI [mL/m^2^]); systemic vascular resistance index (SVRI [dyn*s*cm^−5^*m^2^]); total arterial compliance (TAC [mL*mm Hg^−1^]: TAC = SV*PP^−1^); thoracic fluid content (TFC [1*Ωk^−1^]); velocity index (VI [1000*Z0*s^−1^]: VI = 1000*dZmax*Z0^−1^), which reflects maximum aortic blood flow; Heather index (HI [Ohm*s^2^]): HI = dZmax*TRC), which is an indicator of inotropic cardiac function; and acceleration index (ACI [100*Z0*s^−2^]: ACI = 100*dZmax*dt^−1^), which reflects maximum aortic blood acceleration. In accordance with data analysis from the PREDICT study, we divided the study subjects into different risk groups based on the SI and TFC values, with the adopted cutoff parameters for SI at < 35 mL/m^2^ and for TFC at > 35 1/kOhm [21].

### 2.4. Statistical Methods

The statistical analysis and electronic filing of data were conducted with *MS Office Excel* and *Statistica 12.0* software (StatSoft Inc., Tulsa, OK, USA). The distribution of continuous variables was evaluated using vision and the Shapiro–Wilk test. Continuous variables were presented as means ± standard deviation (SD), interquartile ranges, and medians. In contrast, categorical variables were presented as absolute values (*n*) and proportions (%). In the statistical analysis, propensity score matching was applied to select a special subgroup from the control group matched for the most important clinical criteria (BMI, MBP, sex, age, and the proportion of AH), which may considerably affect the assessed values. The differences in absolute values of continuous variables with a normal distribution were assessed using the t-test, and variables with a non-normal distribution using the Mann–Whiney U-test. The chi-square and Fisher’s exact tests were used to analyse categorical (qualitative) variables. A *p* < 0.05 was considered statistically significant.

## 3. Results

### 3.1. Baseline Characteristics

The demographic and clinical data of the PR group and the CG have been presented in Table 1.

The MBP in the PR group was 116/76 mmHg (90% of individuals had BP < 140/90 mmHg). Eight patients (40%) from the PR group had AH, which was treated medically in all cases. Body weight abnormalities were detected in 90% of PR group patients, with ten patients (50%) obese and eight patients (40%) overweight. Three out of 20 patients (15%) had their T2DM diagnosis confirmed, four patients (20%) had prediabetes (IFG or IGT), whereas 13 patients (65%) had normal glucose tolerance. Out of the diabetic prolactinoma patients two patients were treated with metformin and one with metformin and insulin. Nineteen out of 20 patients from the PR group had functional anterior pituitary lobes. One patient with an invasive lactotrope tumor was diagnosed with TSH deficiency, but this was well-controlled with a stable L-thyroxin dose.

The MBP in CG subjects was 119/76 mmHg (96% of the subjects had BP < 140/90 mmHg). In total, one hundred and twenty CG subjects (77%) were diagnosed with AH. All patients with AH had been on medical treatment for at least 12 months.

Out of the routinely measured parameters, the two groups differed markedly only in creatinine levels, with PR patients showing significantly lower creatinine levels than CG patients (0.80 vs. 0.96 mg/dL, respectively; *p* = 0.001). There were, however, no significant between-group differences in terms of patient age, BMI, HR, SBP, or DBP.

### 3.2. ICG Variables

**Figure 1:**
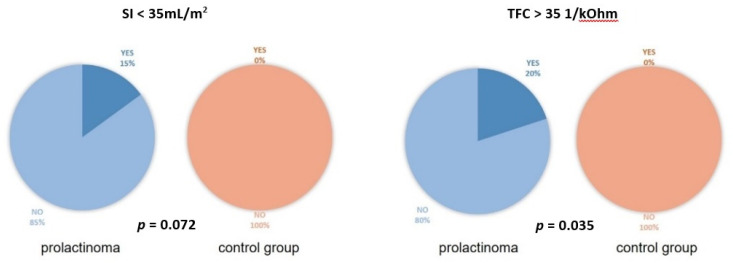
The proportion of low stroke volume index (SI) and high thoracic fluid content (TFC) in the prolactinoma and the control group.

General parameters and ICG variables in the two groups have been presented and compared in Table 2 and in Figure 1.

Three patients (15%) from the PR group showed a low SI of < 35 mL/m^2^. Four patients (20%) from the PR group had an elevated TFC of > 35 1/kOhm) (Figure 1). There were no such abnormal values in the CG.

Notably, PR patients showed lower values of cardiac function parameters (SI and VI), a lower HI, and a higher SVRI than those in the CG group; however, these differences were not statistically significant (Table 2).

## 4. Discussion

This study confirmed the presence of subclinical hemodynamic dysfunction in patients newly diagnosed with prolactinoma, despite optimal BP values in the vast majority of them. A comprehensive hemodynamic function assessment with ICG revealed the hemodynamic profiles of prolactinoma patients are different than those of individuals with no endocrine disorders, with the increased TFC in some individuals being particularly interesting.

The study evaluated male patients newly diagnosed with prolactinoma, with no comorbidities that might have a considerably adverse effect on cardiovascular system function. We would like to emphasize that only subjects with no clinically overt cardiovascular dysfunction were included in this study, whereas patients with severe cardiovascular, respiratory, and neurological conditions that might considerably affect the results were excluded. The cardiac and metabolic risk factors in the study population were comparable with those in another study on concomitant cardiovascular dysfunction in patients with prolactinoma [7]. According to the authors of this study, the incidence of cardiovascular disease is higher only among adult males with prolactinoma.

Although the data on cardiovascular disease rates in patients with prolactinoma are scarce, they unequivocally indicate an increased cardiovascular risk and mortality risk, which is primarily due to metabolic disorders concomitant with the early development of atherosclerosis [7,8,9,10,11,12,13]. This increased risk is associated not so much with PRL oversecretion but rather with the adverse effects of PRL on the hypothalamus-pituitary-gonad axis, which manifest as menstruation abnormalities and abnormal estradiol levels in women and testosterone deficit and hypogonadism-induced metabolic sequelae in men [8].

The results of our study using impedance cardiography—a diagnostic method new to this patient population—showed that patients with prolactinoma tend to have a higher TFC (retain more fluid in the chest) and lower values of cardiac function parameters than in the CG. This may be due to early cardiovascular dysfunction, and abnormalities in balanced fluid distribution. This set of findings in earlier studies identified patients who were at the highest risk of heart failure exacerbation [27]. Among patients with heart failure and hypertension, a correlation has been shown between haemodynamic indices assessed by impedance cardiography and indices of left ventricular function determined by echocardiography [20,21,22,23,24,27]. A significant association has been found between impaired left ventricular systolic function and low values of parameters characterising blood flow assessed by ICG [20,23,27]. Assessment of haemodynamic parameters has also been shown to be clinically relevant in patients with heart failure and preserved left ventricular systolic function [22]. In patients with hypertension, impedance cardiography can be a useful method to assess left ventricular dysfunction, with cardiac output index and systemic vascular resistance being important predictors [20,21,22,24,27].

Our accurate assessment of cardiovascular haemodynamic abnormalities by impedance cardiography is one of the first attempts to apply this method to patients with PR. Such abnormalities may be a result of complex pathological mechanisms that affect the development of cardiovascular complications in patients with prolactinoma. These pathological mechanisms include endothelial dysfunction combined with altered total peripheral resistance and the coexistence of metabolic disorders and insulin resistance [7,8,10,19,28,29]. These observations have important clinical implications due to the fact that long-term PRL secretion-induced cardiovascular complications definitively worsen the unfavorable prognosis in patients with prolactinoma. Studies on this topic unequivocally indicate that patients with prolactinoma have an increased risk of death due to structural and functional changes in the cardiovascular system that lead to cardiovascular remodeling [8,17,19,30].

Early diagnosis of cardiovascular complications in patients with prolactinoma, even prior to clinical manifestations, seems to be clinically significant and may contribute to identifying patients who require closer cardiological monitoring in order to reduce the risk of developing overt cardiovascular disease and death.

Patients with PR and symptomatic heart disease are usually treated with standard guideline-based therapy. However, there is insufficient evidence to make reliable therapeutic recommendations for patients with PR with asymptomatic left ventricular dysfunction. The aforementioned pathomechanisms prompt the use of vasodilator-based polytherapy (angiotensin-converting enzyme inhibitors, calcium blockers) in combination with diuretics for the treatment of hypertension in this patient group. The assessment of subclinical left ventricular dysfunction by impedance cardiography can identify patients who may benefit from appropriate heart disease prevention. Furthermore, the detection of impaired cardiac function may prompt a more intensive therapeutic approach in patients previously treated with hypotensive drugs. Regular clinical, electrocardiographic and echocardiographic examinations should include early signs of heart failure that require a specific therapeutic approach.

Similar conclusions apply to patients with Cushing disease and acromegaly [10,30,31], who were also shown to have characteristic hemodynamic profiles via the use of ICG [32,33].

### Study Limitations

The main limitation of this study was the relatively small sample size. This was due to low incidence of prolactinoma in men. Moreover, we included only those patients with prolactinoma who had no clinically apparent features of cardiovascular dysfunction and excluded patients with serious comorbidities. When interpreting the study results, it is important to consider the potential effect of concomitant AH (despite it being well-controlled), the duration of AH (this was not assessed in detail), and the antihypertensive treatment used. Moreover, the effects of the patients’ sex (females were not evaluated in this study) on hemodynamic dysfunction in active PRL-secreting pituitary tumors requires further study. A further limitation of this study was the fact that three patients with prolactinoma developed diabetes, although all of those patients were effectively treated with low doses of oral antidiabetic agents.

## 5. Conclusions

Prolactinoma-associated endocrine disorders are due to hemodynamic profile differences, including higher rates of increased thoracic fluid content and a tendency towards worse cardiac function. Personalized ICG assessment in patients with prolactinoma may be useful in identifying these abnormalities at an early stage and initiating appropriate therapeutic decisions.

## Figures and Tables

**Table 1:** Baseline characteristics of the patients with prolactinoma.

VARIABLE	Mean ± SD (Median; Interquartile Range) or *n* (%)
Age [years].	43 ± 13 (42; 31–52)
Male sex	20 (100)
BMI [kg/m^2^]	32 ± 7 (30; 28–35)
BMI 18.5–24.9 kg/m^2^	2 (10)
BMI 25–29.9 kg/m^2^	8 (40)
BMI ≥ 30 kg/m^2^	10 (50)
HR [bpm]	68 ± 10 (69; 61–74)
SBP [mmHg]	117 ± 17 (119; 102–130)
SBP ≥ 140 mmHg	2 (10)
DBP [mmHg]	76 ± 10 (77; 66–82)
DBP ≥ 90 mmHg	2 (10)
AH	8 (40)
DM	3 (15)
Creatinine [mg/dL]	0.80
LVEF [%]	64 ± 3 (64; 63–65)

AH—arterial hypertension; BMI—body mass index; DBP—diastolic blood pressure; DM—diabetes mellitus; HR—heart rate; LVEF—left ventricular ejection fraction; RAAB—renin angiotensin aldosterone blockers; SBP—systolic blood pressure; SD—standard deviation.

**Table 2:** The comparison of routinely evaluated parameters and the hemodynamic parameters measured using impedance cardiography in the control group (CG) and the group of patients with prolactinoma (PR).

VARIABLES	CG Mean ± SD (Median; IQR) or *n* (%)	PR Mean ± SD (Median; IQR) or *n* (%)	*p*-Value
**BASELINE CHARACTERISTICS**			
Age [years]	43 ± 9 (43; 37–50)	43 ± 13 (42; 31–52)	0.922
BMI [kg/m^2^]	30 ± 4 (29; 27–31)	32 ± 7 (30; 28–35)	0.525
HR [bpm]	66 ± 10 (67; 59–74)	68 ± 10 (69; 61–74)	0.692
SBP [mmHg]	119 ± 7 (117; 114–123)	117 ± 17 (119; 102–130)	0.567
DBP [mmHg]	75 ± 8 (74; 69–81)	76 ± 10 (77; 66–82)	0.684
AH	11/20 (55)	8/20 (40)	0.342
Creatinine [mg/dL]	0.96 ± 0.14 (0.94; 0.90–1.00)	0.80 ± 0.13 (0.80; 0.70–0.85)	0.001
LVEF [%]	66 ± 3 (66; 64–68)	64 ± 3 (64; 63–65)	0.051
**IMPEDANCE CARDIOGRAPHY**			
HR [bpm]	67 ± 13 (68; 58–74)	67 ± 10 (67; 61–72)	0.989
SBP [mmHg]	117 ± 9 (117; 109–124)	116 ± 16 (119; 102–131)	0.682
DBP [mmHg]	75 ± 7 (75; 73–80)	76 ± 10 (78; 66–82)	0.762
MBP [mmHg]	85 ± 7 (85; 81–91)	86 ± 11 (89; 76–92)	0.873
PP [mmHg]	43 ± 8 (43; 38–48)	40 ± 9 (39; 35–46)	0.355
SI [mL/m^2^]	50 ± 11 (53; 41–58)	47 ± 11 (45; 40–54)	0.301
CI [mL*m^−2^*min^−1^]	3.3 ± 0.7 (3.4; 2.9–3.8)	3.1 ± 0.6 (3.0; 2.7–3.4)	0.211
SVRI [dyn*s*cm^−5^*m^2^]	1994 ± 498 (1871; 1721–2228)	2127 ± 402 (2091; 1811–2378)	0.359
VI [1*1000^−1^*s^−1^]	46 ± 13 (43; 35–60)	43 ± 12 (43; 36–50)	0.400
ACI [1/100/s^2^]	71 ± 25 (72; 49–95)	67 ± 22 (70; 53–81)	0.640
HI [Ohm/s^2^]	12 ± 3 (12; 10–15)	10 ± 3 (10; 8–12)	0.112
TFC [1/kOhm]	30 ± 4 (30; 28–32)	30 ± 4 (31; 27–33)	0.732

ACI—acceleration index; BMI—body mass index; CI—cardiac index; DBP—diastolic blood pressure; HI—Heather index; HR—heart rate; IQR—interquartile range; MBP—mean blood pressure; PP—pulse pressure; SBP—systolic blood pressure; SD—standard deviation; SI—stroke index; SVRI—systemic vascular resistance index; TAC—total artery compliance; TFC—thoracic fluid content; VI—velocity index.

## Data Availability

Not applicable.

## References

[1] Casanueva F.F., Molitch M.E., Schlechte J.A., Abs R., Bonert V., Bronstein M.D., Brue T., Cappabianca P., Colao A., Fahlbusch R. (2006). Guidelines of the Pituitary Society for the diagnosis and management of prolactinomas. Clin. Endocrinol..

[2] Melmed S., Casanueva F.F., Hoffman A.R., Kleinberg D.L., Montori V.M., Schlechte J.A., Wass J.A. (2011). Endocrine Society. Diagnosis and treatment of hyperprolactinemia: An Endocrine Society clinical practice guideline. J. Clin. Endocrinol. Metab..

[3] Daly A.F., Beckers A. (2020). The Epidemiology of Pituitary Adenomas. Endocrinol. Metab. Clin..

[4] Fernandez A., Karavitaki N., Wass J.A. (2010). Prevalence of pituitary adenomas: A community-based, cross-sectional study in Banbury (Oxfordshire, UK). Clin. Endocrinol..

[5] Raappana A., Koivukangas J., Ebeling T., Pirilä T. (2010). Incidence of pituitary adenomas in Northern Finland in 1992–2007. J. Clin. Endocrinol. Metab..

[6] Gruppetta M., Mercieca C., Vassallo J. (2013). Prevalence and incidence of pituitary adenomas: A population based study in Malta. Pituitary.

[7] Toulis K.A., Robbins T., Reddy N., Balachandran K., Gokhale K., Wijesinghe H., Cheng K.K., Karavitaki N., Wass J., Nirantharakumar K. (2018). Males with prolactinoma are at increased risk of incident cardiovascular disease. Clin. Endocrinol..

[8] Krogh J., Selmer C., Torp-Pedersen C., Gislason G.H., Kistorp C. (2017). Hyperprolactinemia and the Association with All-Cause Mortality and Cardiovascular Mortality. Horm. Metab. Res..

[9] Pala N.A., Laway B.A., Misgar R.A., Dar R.A. (2015). Metabolic abnormalities in patients with prolactinoma: Response to treatment with cabergoline. Diabetol. Metab. Syndr..

[10] dos Santos Silva C.M., Barbosa F.R., Lima G.A., Warszawski L., Fontes R., Domingues R.C., Gadelha M.R. (2011). BMI and metabolic profile in patients with prolactinoma before and after treatment with dopamine agonists. Obesity.

[11] Berinder K., Nyström T., Höybye C., Hall K., Hulting A.L. (2011). Insulin sensitivity and lipid profile in prolactinoma patients before and after normalization of prolactin by dopamine agonist therapy. Pituitary.

[12] Arslan M.S., Topaloglu O., Sahin M., Tutal E., Gungunes A., Cakir E., Ozturk I.U., Karbek B., Ucan B., Ginis Z. (2014). Preclinical atherosclerosis in patients with prolactinoma. Endocr. Pract..

[13] Erem C., Kocak M., Nuhoglu I., Yılmaz M., Ucuncu O. (2010). Blood coagulation, fibrinolysis and lipid profile in patients with prolactinoma. Clin. Endocrinol..

[14] Ciccarelli A., Daly A.F., Beckers A. (2005). The epidemiology of prolactinomas. Pituitary.

[15] Reuwer A.Q., van Zaane B., van Wissen M., van Zanten A.P., Twickler M.T., Gerdes V.E. (2011). Prolactin is involved in the systemic inflammatory response in myocardial infarction. Horm. Metab. Res..

[16] Punjabi N.M., Shahar E., Redline S., Gottlieb D.J., Givelber R., Resnick H.E. (2004). Sleep Heart Health Study Investigators. Sleep-disordered breathing, glucose intolerance, and insulin resistance: The Sleep Heart Health Study. Am. J. Epidemiol..

[17] Reuwer A.Q., Twickler M.T., Hutten B.A., Molema F.W., Wareham N.J., Dallinga-Thie G.M., Bogorad R.L., Goffin V., Smink-Bol M., Kastelein J.J. (2009). Prolactin levels and the risk of future coronary artery disease in apparently healthy men and women. Circ. Cardiovasc. Genet..

[18] Jiang X.B., Zhang J., Li C.L., Mao Z.G., Hu B., Zhu Z., Zhu Y.H., Wang H.J. (2017). Subclinical impairment of left ventricular longitudinal function in patients with prolactinomas. Endocr. Pract..

[19] Reuwer A.Q., Sondermeijer B.M., Battjes S., van Zijderveld R., Stuijver D.J., Bisschop P.H., Twickler M.T., Meijers J.C., Schlingemann R.O., Stroes E.S. (2012). Microcirculation and atherothrombotic parameters in prolactinoma patients: A pilot study. Pituitary.

[20] Krzesiński P., Gielerak G., Kowal J. (2009). Impedance cardiography—A modern tool for monitoring therapy of cardiovascular diseases. Kardiol. Pol..

[21] Bhalla V., Isakson S., Bhalla M.A., Lin J.P., Clopton P., Gardetto N., Maisel A.S. (2005). Diagnostic ability of B-type natriuretic peptide and impedance cardiography: Testing to identify left ventricular dysfunction in hypertensive patients. Am. J. Hypertens..

[22] Krzesiński P., Gielerak G., Stańczyk A., Uziębło-Życzkowska B., Smurzyński P., Piotrowicz K., Skrobowski A. (2015). What does impedance cardiography add more to the assessment of left ventricular diastolic function in essential hypertension?. Pol Merkur Lek..

[23] Parrott C.W., Burnham K.M., Quale C., Lewis D.L. (2004). Comparison of changes in ejection fraction to changes in impedance cardiography cardiac index and systolic time ratio. Congest. Heart Fail..

[24] Abdelhammed A.I., Smith R.D., Levy P., Smits G.J., Ferrario C.M. (2005). Noninvasive hemodynamic profiles in hypertensive subjects. Am. J. Hypertens..

[25] Fleseriu M., Hashim I.A., Karavitaki N., Melmed S., Murad M.H., Salvatori R., Samuels M.H. (2016). Hormonal Replacement in Hypopituitarism in Adults: An Endocrine Society Clinical Practice Guideline. J. Clin. Endocrinol. Metab..

[26] Williams B., Mancia G., Spiering W., Agabiti Rosei E., Azizi M., Burnier M., Clement D.L., Coca A., de Simone G., Dominiczak A. (2018). ESC Scientific Document Group. 2018 ESC/ESH Guidelines for the management of arterial hypertension. Eur. Heart J..

[27] Strobeck J.E., Silver M.A. (2004). Beyond the four quadrants: The critical and emerging role of impedance cardiography in heart failure. Congest. Heart Fail..

[28] Friedrich N., Schneider H.J., Spielhagen C., Markus M.R., Haring R., Grabe H.J., Buchfelder M., Wallaschofski H., Nauck M. (2011). The association of serum prolactin concentration with inflammatory biomarkers—Cross-sectional findings from the population-based Study of Health in Pomerania. Clin. Endocrinol.

[29] Haring R., Friedrich N., Völzke H., Vasan R.S., Felix S.B., Dörr M., Meyer zu Schwabedissen H.E., Nauck M., Wallaschofski H. (2014). Positive association of serum prolactin concentrations with all-cause and cardiovascular mortality. Eur. Heart J..

[30] Melmed S. (2006). Medical progress: Acromegaly. N. Engl. J. Med..

[31] Pivonello R., Auriemma R.S., Grasso L.F., Pivonello C., Simeoli C., Patalano R., Galdiero M., Colao A. (2017). Complications of acromegaly: Cardiovascular, respiratory and metabolic comorbidities. Pituitary.

[32] Jurek A., Krzesiński P., Gielerak G., Witek P., Zieliński G., Kazimierczak A., Wierzbowski R., Banak M., Uziębło-Życzkowska B. (2021). Cushing’s Disease: Assessment of Early Cardiovascular Hemodynamic Dysfunction With Impedance Cardiography. Front. Endocrinol..

[33] Jurek A., Krzesiński P., Gielerak G., Witek P., Zieliński G., Kazimierczak A., Wierzbowski R., Banak M., Uziębło-Życzkowska B. (2022). Acromegaly: The Research and Practical Value of Noninvasive Hemodynamic Assessments via Impedance Cardiography. Front. Endocrinol..

